# Mycelial culture extracts of selected wood-decay mushrooms as a source of skin-protecting factors

**DOI:** 10.1007/s10529-021-03095-0

**Published:** 2021-02-23

**Authors:** Katarzyna Sułkowska-Ziaja, Karolina Grabowska, Anna Apola, Agata Kryczyk-Poprawa, Bożena Muszyńska

**Affiliations:** 1 Faculty of Pharmacy, Department of Pharmaceutical Botany, Jagiellonian University Medical College, Medyczna 9, 30-688 Kraków, Poland; 2 Faculty of Pharmacy, Department of Pharmacognosy, Jagiellonian University Medical College, Medyczna 9, 30-688 Kraków, Poland; 3 Faculty of Pharmacy, Department of Inorganic Chemistry, Jagiellonian University Medical College, Medyczna 9, 30-688 Kraków, Poland

**Keywords:** *Ganoderma applanatum*, Hyaluronidase, Inhibition, *Laetiporus sulphureus*, Sun protection factor, *Trametes versicolor*, Tyrosinase

## Abstract

**Objectives:**

This study analyzed the content of substances with cosmetologic properties in the extracts obtained from the mycelial cultures of *Ganoderma applanatum*, *Laetiporus sulphureus*, and *Trametes versicolor*. The effect of these extracts on the inhibition of tyrosinase and hyaluronidase was determined, and their values of sun protection factor (SPF) were calculated.

**Results:**

The total amount of phenolic acids in the extracts ranged from 2.69 (*G. applanatum*) to 10.30 mg/100 g dry weight (*T. versicolor*). The total amount of sterols was estimated at 48.40 (*T. versicolor*) to 201.04 mg/100 g dry weight (*L. sulphureus*), and that of indoles at 2.90 (*G. applanatum*) to 16.74 mg/100 dry weight (*L. sulphureus*). Kojic acid was determined in the extracts of *L. sulphureus* and *G. applanatum*. It was observed that *L. sulphureus* extract caused dose-dependent inhibition of hyaluronidase, while all the extracts inhibited tyrosinase. The extract of *G. applanatum* exhibited an SPF value of ~ 9.

**Conclusions:**

The results showed that the mycelial cultures of the studied species may be used as an alternative source of substances used in cosmetology.

## Introduction

Mushrooms are well known for their therapeutic properties for thousands of years. Since the mid-twentieth century, intensive research has been undertaken on the bioactive compounds present in fruiting bodies. Nowadays, studies are more frequently conducted with the use of biotechnological methods. Similar to fruiting bodies, mycelial cultures constitute a source of bioactive compounds, such as polysaccharides, phenolic compounds, terpenes, sterols, or fatty acids. The extracts obtained from the mycelial biomass have been proven to possess anticancer, antioxidant, antimicrobial, and anti-inflammatory properties (Smith et al. 2002).

The data on Traditional Chinese Medicine and modern studies have demonstrated the possibility of using fruiting bodies and mycelial cultures as the source of substances that are applied in cosmetology. Due to their high antioxidant potential, fungal extracts have been utilized in antiaging preparations, and due to the presence of anti-inflammatory or antimicrobial compounds, they are more commonly used, in the preparation for problematic skin. In addition, fungal extracts possess whitening, moisturizing, and nutritional effects and have the ability to absorb ultraviolet rays, and so they can be used as a natural sunscreen (Hyde et al. 2010).

The present study analyzed the mycelial cultures of three wood-decay mushroom species, namely *Ganoderma applanatum*, *Laetiporus sulphureus*, and *Trametes versicolor*. The biological properties of the compounds present in these species were widely determined. Among the studied species, *L. sulphureus* has been shown to exhibit, for example, anti-inflammatory activity which could be attributed to the presence of exopolysaccharides (Jayasooriya et al. 2011). The cytotoxic properties of this species are associated with terpenes (Léon et al. 2004). PSK (protein and polysaccharide complex) isolated from *T. versicolor* is used in Japan as an adjuvant in cancer therapy and is considered as the first confirmed drug of fungal origin (Sakagami et al. 1991). Triterpenes found in *Ganoderma* sp. are responsible for the anticancer or antioxidant effects (Chairul and Hayashi 1994).

This study is the first to analyze the bioactive secondary metabolites from mycelial cultures in terms of their antiaging properties. The content of phenolic acids, sterols, indole derivatives, and kojic acid in the mycelial extracts was determined by reverse-phase high-performance liquid chromatography (HPLC) with diode array detection. The antityrosinase and antihyaluronidase activities of the extracts were investigated in vitro. In addition, the sun protection factor (SPF) values of the extracts were calculated.

## Materials and methods

### Mycelial cultures

The materials used for establishing the mycelial cultures analyzed in the study were the fruiting bodies of *G. applanatum* (Pers.) Pat., *L. sulphureus* (Bull.) Murrill, *T. versicolor* (L.) Lloyd, which were collected in September 2015 from the forests of southern Poland (Krynica forest district, Małopolskie voivodeship).

The initial cultures established on Petri dishes, on the medium prepared according to Oddoux (1957), were incubated at 22 ± 2 °C in a thermostat (ST500/B/40; POL-EKO-APARATURA, Poland) without access to light. The voucher specimens of *G. applanatum* GA/I/KBF/UJCM/21015, *L. sulphureus* LS/I/KBF/UJCM/21015, and *T. versicolor* TV/I/KBF/UJCM/21015 and the fruiting body samples are stored at the Department of Pharmaceutical Botany of the Jagiellonian University Medical College.

The inoculum used for experimental cultures was mycelial fragments taken from the initial cultures on the solid medium. The experimental cultures were carried out in 300-mL Erlenmeyer flasks containing 100 mL of liquid medium and were shaken at 140 rpm (ALTEL, Łódź, Poland). They were maintained at 22 ± 2 °C with a variable photoperiod, depending on the season. After a 3-week growth cycle (three culture series in three repetitions), the biomass was separated from the medium, frozen, lyophilized (Labconco, USA), and stored at 25 ± 2 °C.

### Estimation of phenolic acids

The powdered material (2.0 g dry weight – DW) of each sample was extracted with 50 mL of boiling methanol for 2 h in a reflux condenser. The obtained extract was concentrated (Rotavapor R-114; Büchi, Germany) to dry. Dry residue was dissolved in 2 mL of HPLC-grade methanol. The qualitative and quantitative analyses of the residue were performed according to Ellnain-Wojtaszek and Zgórka (1999) with some modifications. The HPLC analysis, equipment used, and analysis conditions were the same as those described in our previous study (Sułkowska-Ziaja et al. 2018).

### Estimation of sterols

The powdered material (2.0 g DW) of each sample was extracted with a methanol/dichloromethane mixture (75:25, v/v) using ultrasound at a frequency of 49 kHz for 20 min (Sonic-2; Polsonic, Poland). After 2 h, the extract was centrifuged for 15 min at 4500 rpm (MPW 342; MPW Med. Instruments, Poland). Then, the supernatant was collected and the procedure was repeated. The obtained extracts were combined and evaporated to dryness at 22 ± 2 °C (Rotavapor R-114; Büchi, Germany). The dry residue was dissolved in 2 mL of HPLC-grade methanol. The qualitative and quantitative analyses of the residue were carried out following the procedure developed by Yuan et al. (2006) with some modifications in the gradient procedure. The HPLC analysis, equipment used, and analysis conditions were the same as those described in our previous study (Sułkowska-Ziaja et al. 2018).

### Estimation of indole derivatives

The powdered material (2.0 g DW) of each sample was extracted 10 times with portions of methanol (10 mL) using ultrasound (49 kHz) for 20 min (Sonic-2; Polsonic, Poland). The obtained extracts were combined and evaporated to dryness at 22 ± 2 °C (Rotavapor R-114; Büchi, Germany). The dry residues were dissolved in 2 mL of HPLC-grade methanol. The HPLC analysis, equipment used, and analysis conditions were the same as those previously described by Muszyńska et al. (2011).

### Estimation of kojic acid

The powdered material (0.5 g DW) of each sample was extracted with 50% methanol. After 4 h, the extract was centrifuged for 15 min at 4500 rpm (MPW 342; MPW Med. Instruments, Poland). Then, the supernatant was collected and the residue was subjected to repeated extraction. The obtained supernatants were combined and evaporated to dryness at 22 ± 2 °C (Rotavapor R-114; Büchi, Germany). The dry residue was dissolved in 2 mL of methanol with HPLC-grade. The HPLC analyses were performed using a Merck-Hitachi apparatus. The detection was set at 270 nm. A Purospher® RP-18e column (250 × 4 mm, 5 µm; Merck) was used at 35 °C. Isocratic elution was applied according to Kimura et al. (2000). The mobile phase used was composed of 0.1 M sodium dihydrogen/methanol (97:3, v/v). The flow rate was set at 1 mL/min, and the injection volume was 10 µL.

### Qualitative and quantitative analysis

The analyzed compounds were identified by comparing their retention times with those of the reference compounds and by applying co-chromatography with standards. Besides, the UV spectra of the respective peaks were compared with that of the model compounds. Quantitative analysis was performed using the calibration curve method, by assuming a linear relationship between the surface of the field under the curve and the concentration of the examined compounds. Quantification was done by measuring the peak areas with reference to the standard curve derived from five concentrations (0.03125–0.5 mg/mL). The quantitative results were expressed in mg/100 g DW as mean ± SD from three series of tests, which were repeated three times.

### Inhibition of tyrosinase

The influence of extracts on the inhibition of tyrosinase was assessed using the method developed by Chien et al. (2008). The degree of inhibition was determined by spectrophotometry from the UV spectrum with modifications. The percentage of inhibition was estimated by measuring the absorbance of the tested mixtures at a 274 nm. At the same time, the spectra of the entire UV range were monitored to identify any undesirable absorption spectrum that could indicate additional reactions in the analyzed mixtures. The evaluation was carried out in a phosphate buffer solution with pH 6.84, in which the analyzed extracts were dissolved. The prepared tyrosinase control solution contained 350 units of enzyme in 1.0 mL. The inhibiting activity was expressed as a percentage of enzyme inhibition, and the assessment of the force of action was carried out relative to kojic acid. The analyzed mixtures were incubated at 37 °C for 25 min. Subsequently, an absorption spectrum was obtained within the wavelength range of 200–400 nm in which a well-developed maximum was observed at 274 nm. The absorption at the maximum was used to evaluate the inhibiting action of the analyzed extracts or kojic acid on tyrosinase activity. The impact of the extracts on the enzyme activity was determined using the formula: (%inhibition) = [1 − (B − B0)/(A − A0)] × 100, where A is the absorbance of the control sample (with tyrosinase, l = 274 nm), A0 is the absorbance of the blank sample (without tyrosinase, l = 274 nm), B is the absorbance of the test sample (with tyrosinase, l = 274 nm), and B0 is the absorbance of the test sample (without tyrosinase, l = 274 nm).

### Inhibition of hyaluronidase

The impact of extracts on hyaluronidase inhibition was assessed using the turbidimetric method developed by Di Ferrante (1956) with minor modifications (Grabowska et al. 2016). This method is based on the formation of insoluble complexes between nonhydrolyzed hyaluronic acid (HA) and cetyltrimethylammonium bromide (CTAB). The test was adjusted to performance using microplates. First, the analyzed extracts were dissolved in methanol. The reaction mixtures were prepared by mixing 10 µL of the test sample, 25 µL of incubation buffer (acetate buffer pH 4.5 with 0.5 mg/mL of albumin and 77 mM NaCl), 15 µL of acetate buffer (pH 4.5), and 25 µL of the enzyme (Hyal, 30 U/mL). The prepared reaction mixtures were subjected to preincubation (10 min, 37 °C). Then, 25 µL of HA solution (0.3 mg/mL) was added, and the mixtures were subsequently incubated (45 min, 37 °C). Following incubation, 2.5% CTAB solution was added to the reaction mixtures. The inhibitory effect of extracts on enzyme activity was assessed by measuring the absorbance of the precipitated solution, nonhydrolyzed hyaluronic acid, as a complex with CTAB. The absorbance was measured using a Multi-Detection Microplate Reader (Synergy HT; BioTek) at a 600 nm. The tested samples were analyzed at a final concentration of 0.1–3 mg/mL. The inhibiting activity was expressed as a percentage of enzyme inhibition, and the assessment of the force of action was carried out relative to the reference substance, quercetin. During the analysis, control tests were performed by measuring absorbance in the presence of enzyme and substrate (control I) and without the enzyme (control II). Blank samples, which contained only medium (blank sample of the experiment) or substances at the tested concentration (product control), were analyzed in parallel. All the determinations were performed in three repetitions. The percentage of inhibition was calculated based on the formula: %inhibition = {[As–(APc–AB)]–AI}/{[AII–(APc–AB)] − AI} × 100, where AI is the absorbance in the presence of enzyme and substrate (control I), AII is the absorbance of sample without enzyme (control II), AS is the absorbance of the test sample, APc is the absorbance of the product control solution, and AB is the absorbance of a blank control of the experiment.

### In vitro assay to determine the SPF

The powdered material (0.5 g DW) of each sample was extracted with ethanol. Then, 100 mg of dry ethanolic extracts was weighted, transferred into volumetric flasks, and ethanol was added to make up to a final volume of 10 mL. The samples were left for 24 h followed by ultrasonication in an ultrasonic bath for 10 min. Subsequently, they were filtered and properly diluted with ethanol, to a final concentration of 200 μg/mL. The absorbance values of the investigated arboreal fungi solutions were determined from 290 to 320 nm, at 5-nm intervals, using ethanol as blank, in a Cary 100 Spectrophotometer (Varian, USA) equipped with 1-cm quartz cells. The SPF of the arboreal fungi extracts was calculated by applying the Mansur mathematical equation (Mansur et al. 1986):

$$ SPF_{in vitro} = CF \times \mathop \sum \limits_{290}^{320} EE\left( \lambda \right) \times I\left( \lambda \right) \times abs\left( \lambda \right) $$

where CF is the correction factor (10) and

$$ \mathop \sum \limits_{290}^{320} EE\left( \lambda \right) \times I\left( \lambda \right) \times abs\left( \lambda \right) $$

is the sum of absorbances at the specified interval, weighted with the values of $EE\left(\lambda \right)\times I\left(\lambda \right)$ which are constant (Sayre et al. 1979).

### Statistical analysis

All the experiments were repeated three times. Data are presented as mean ± SD. Statistical analysis was performed using one-way analysis of variance with Tukey’s post hoc method. The values of p < 0.05 were considered statistically significant.

## Results and discussion

### Mycelial cultures

The extracts of plant origin used as active components with a broad spectrum of action are of high value in cosmetology. However, in the search for new solutions and cosmetic products of better quality, the cosmetic industry commonly uses extracts from fungi, which constitute a rich source of active substances. Basidiomycota species, which have long been used to treat various ailments including dermal disorders, have become an inspirations for formulating products that can prevent and alleviate the skin aging process, reduce inflammation, and nourish and regenerate the skin (Hyde et al. 2010; Taofiq et al. 2016).

The possibility of maintaining mycelial cultures made them comfortable alternatives to materials obtained from natural habitats. In the present study, the mycelial cultures of *G. applanatum*, *L. sulphureus*, and *T. versicolor* were set up within the experiment. After 7 days, the mycelium was observed in the form of colonies covering the surface of the medium. The obtained mycelia were microscopically analyzed, which indicated the homogeneous characteristics of the hyphae and the absence of contamination by other fungal or bacterial strains. Within the experimental cultures, the growth of mycelium was observed as spherical, compact aggregates with a bright cream color (*G. applanatum*, *T. versicolor*) or yellow and orange color (*L. sulphureus*). For all the culture series conducted on the liquid medium, the mean values of the biomass growth parameter were determined as dry weight from 1 L of the medium: *G. applanatum*—15.96 ± 0.98 g/L, *L. sulphureus*—7.064 ± 1.48 g/L, and *T. versicolor—*6.778 ± 2.01 g/L.

### Chemical analysis

The analysis of the qualitative composition of the extracts indicated that among 20 analyzed compounds (19 phenolic acids and cinnamic acid), three were present in all the extracts of the investigated species—gallic, protocatechuic, and *p*-hydroxybenzoic acid. In addition, syringic acid was identified in *G. applanatum*, and gentisic acid in *T. versicolor.* The highest amount of phenolic compounds was detected in the extracts of *T. versicolor*—10.30 mg/100 g DW. The amounts of individual compounds ranged from 0.07 (gallic acid in *T. versicolor* extract) to 4.99 mg/100 g DW (*p*-hydroxybenzoic acid in *T. versicolor* extract). The results of the chemical analysis of phenolic acids are shown in Table 1.

**Table 1:** Quantitative comparison of the studied groups of compounds in the mycelial cultures of *Ganoderma applanatum* (Ga), *Laetiporus sulphureus* (Ls), and *Trametes versicolor* (Tv)

Names of compounds	*Ganoderma applanatum*	*Laetiporus sulphureus*	*Trametes versicolor*
	Amounts of compounds [mg/100 g DW]		
Phenolic acids			
Gallic acid	0.89 ± 0.23^b,c^	0.39 ± 0.15^a^	0.07 ± 0.49^a^
Protocatechuic acid	0.93 ± 0.15^b,c^	3.29 ± 1.02^a^	4.23 ± 0.77^a^
*p*–Hydroxybenzoic acid	0.80 ± 0.37^b,c^	4.07 ± 0.73^a^	4.99 ± 0.98^a^
Gentisic acid	nd	nd	1.01 ± 0.03
Syringic acid	0.02 ± 0.01	nd	nd
Total amounts	2.69 ± 0.44	7.75 ± 1.79	10.30 ± 2.03
Sterols			
Ergosterol	63.94 ± 3.54^bc^	136.87 ± 8.18^ac^	33.11 ± 3.55^ab^
Ergosterol peroxide	20.47 ± 1.89^b^	64.01 ± 6.01^a^	15.29 ± 1.84
α–Tocopherol	nd	0.16 ± 1.21	nd
Total amounts	84.41 ± 23.94	7.75 ± 1.79	10.30 ± 2.03
Indole derivatives			
l-Tryptophan	1.76 ± 0.58^b^	14.08 ± 1.66^a^	3.91 ± 1.06
Tryptamine	1.12 ± 0.83^ns^	1.16 ± 1.14^ns^	1.69 ± 0.59^ns^
Melatonin	0.021 ± 0.03^ns^	nd	0.01 ± 0.01^ns^
5-OH-l-tryptophan	nd	1.50 ± 0.45	0.90 ± 0.20
Total amounts	2.9 ± 0.90	16.74 ± 6.4	6.51 ± 1.60
Kojic acid	0.014 ± 0.0092^ns^	0.023 ± 0.01^ns^	nd

Data are presented as the mean ± SD (standard deviation); n = 3 repetitions. Tukey test (Statsoft STATISTICA) was used to reveal the differences between paired groups of elements in row. The same letters mean statistically significant differences between species (a = differences between *Ga* and *Ls*, b = differences between *Ga* and *Tv*, c = differences between *Ls* and *Tv*) for p values at least < 0.01; ns = not statistically significant

Phenolic acids constitute the majority of phenolic compounds in fungi (Ferreira et al. 2009; Karaman et al. 2010). The most common phenolic acids found in mushrooms are *p*-hydroxybenzoic, gallic, protocatechuic, *p*-coumaric, vanillic, ferulic, gentisic, and caffeic acid. *t*-Cinnamic acid, which is a compound with a similar structure as phenolic acids, is also found in mushrooms. The less common phenolic acids in fungi are syringic, chlorogenic, ellagic, and veratric acid. In the case of wood-decay mushrooms, protocatechuic and gallic acid are the most commonly found (Karaman et al. 2010). In the study of Barros et al. (2009) the amount of phenolic acids was shown to range from approximately 2.263 mg/100 g DW in the fruiting bodies of *Lactarius deliciosus* to approximately 35.67 mg/100 g DW in *Ramaria botrytis. p*-Hydroxybenzoic acid was the predominant phenolic acid in quantitative terms as it was found in the highest number of the studied species, with the largest amount observed in *Agaricus silvicola* (23.87 mg/100 g DW). On the other hand, protocatechuic, vanillic, and *p*-coumaric acid were found only in a few species. In *Cantharellus cibarius*, *Lycoperdon perlatum*, and *Macrolepiota procera*, only cinnamic acid was found (1.497, 1.436, and 2.153 mg/100 g DW, respectively). In fungi, phenolic acids exhibit a multidirectional therapeutic effect. For example, gallic acid is distinguished by its anticancer, astringent, antioxidative, antisweat, and antimicrobial effects, and hence has been commonly applied in cosmetology. Protocatechuic acid possesses fungicidal, antihelmintic, and antioxidative properties, while *p*-hydroxybenzoic acid exhibits antibacterial and fungicidal activities and is thus added as a preservative in cosmetic products (Hyde et al. 2010).

The experiments conducted in the present study proved the potential accumulation of phenolic acids in the mycelial cultures of selected wood-decay mushrooms. However, researches on the accumulation of secondary metabolites in mycelial cultures are limited. Further studies on in vitro cultures may prove useful in optimizing the conditions for mycelial cultures and thus increasing the accumulation of phenolic acids.

The qualitative analysis of sterols demonstrated the presence of ergosterol and ergosterol peroxide in the extracts obtained from the biomass of all the studied species (Table 1). In addition, α-tocopherol was detected in the extract from *L. sulphureus* biomass. The amounts of individual metabolites varied among the species and ranged from 0.16 (α-tocopherol in the biomass of *L. sulphureus* mycelial culture) to 136.87 mg/100 g DW (ergosterol in *L. sulphureus *in vitro culture). The total amount of sterols in the biomass was estimated at 48.40 (*T. versicolor*) to 201.04 mg/100 g DW (*L. sulphureus*).

Sterols are commonly found compounds in the fungal kingdom (Weete 2010). As natural components of cellular membranes, these compounds are a subject of interest for the producers of cosmetic preparations. Sterols can penetrate through the corneal layer of the epidermis, and regulate the transepidermal water loss, supplement the protective barrier lipids of the skin, and increase the flexibility of the epidermis. They are also responsible for proper moisturizing of the skin and prevent its premature aging. In addition, these compounds fulfill an essential function in cosmetic preparations, as they act as emulsifiers in water-in-oil emulsion systems (Lodén et al. 1992).

The analyses of fruiting bodies conducted so far have proven that ergosterol is the most common sterol compound. Due to its antibacterial and anti-inflammatory activities, and ability to accelerate the wound healing process, ergosterol is used in preparations formulated for acne and irritated skin, as well as for dry, damaged skin requiring moisturizing and regeneration.

Our previous research showed that the total amount of sterols in the fruiting bodies of *G. applanatum* and *T. versicolor* was 500.88 and 318.83 mg/100 g DW, respectively (unpublished). The experiments conducted in the present study proved the capacity of sterol accumulation in the mycelial cultures of wood-decay mushrooms.

**Fig. 1:**
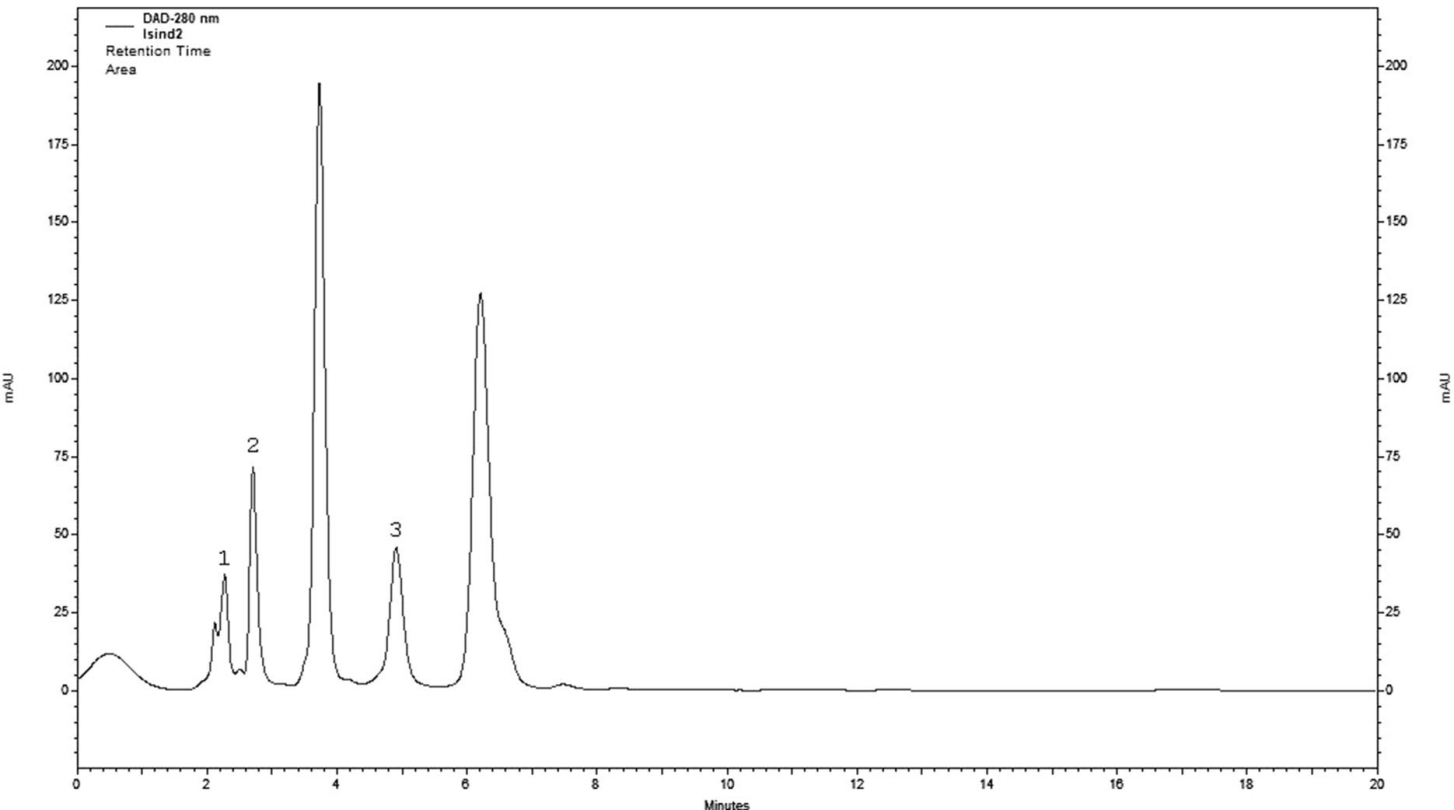
An example HPLC chromatogram—separation of indole compounds in the extract obtained from the in vitro cultures of *Laetiporus sulphureus*: (1) l-tryptophan; (2) tryptamine; and (3) 5-OH-l-tryptophan

The qualitative analysis conducted in this study revealed the presence of four indole derivative compounds of nonhallucinogenic nature. The common metabolites found in all the tested extracts were tryptamine and l-tryptophan. Besides, melatonin was detected in the biomass of *G. applanatum* and *T. versicolor*, and 5-OH-l-tryptophan was found in the biomass of *L. sulphureus* and *T. versicolor*. l-tryptophan was predominant in quantitative terms in the methanol extract obtained from the mycelial culture of *L. sulphureus* at an amount of 14.08 mg/100 g DW. The total amount of indole derivatives ranged from 2.09 (*G. applanatum*) to 16.74 mg/100 g DW (*L. sulphureus*) (Table 1). Figure 1 shows an exemplary HPLC chromatogram for the indole compounds separated in the methanolic extract obtained from the mycelial culture of *L. sulphureus*.

Indole derivatives play a significant role in maintaining the homeostasis of organisms. They influence the function of the nervous system, acting as the function of neurotransmitters and their precursors. In higher fungi, these compounds are represented by tryptophan and tryptamine derivatives (Muszyńska and Sułkowska-Ziaja 2015). Our previous study proved their occurrence in the fruiting bodies of *Phellinus* spp. (Sułkowska-Ziaja et al. 2017) and in the mycelial cultures of *Fomitopsis betulina* (Sułkowska-Ziaja et al. 2018).

Numerous studies have shown the presence of indole derivatives in the fruiting bodies and mycelial cultures of various edible mushroom species. These compounds have been used as pigments in make-up cosmetics and as filters for protecting against UV radiation—one of the critical factors affecting the skin aging process. The present study proved for the first time that the in vitro cultures of the studied species can accumulate indole derivatives.

Our preceding research demonstrated that the amount of indole compounds in the fruiting bodies of *L. sulphureus* and *T. versicolor* was 1.24 and 0.87 mg/100 g DW, respectively. The obtained results confirmed that the fruiting bodies of these species are characterized by a lower amount of indole compounds than the in vitro cultures.

### Antityrosinase and antihyaluronidase activity of the extracts

An increase in the concentration of the studied extracts was found to result in increased inhibition of tyrosinase. However, in the case of *L. sulphureus* extract, an increase in the concentration of the extract from 1000 µg did not increase the inhibition level.

The results of the enzyme inhibition analysis are presented in Table 2. Among the whitening substances, tyrosinase inhibitors comprise the major group widely used in therapy. Tyrosinase controls the rate at which melanin is formed, and thus, many of the substances used to lighten up the skin act through modifying its activity. Inhibition of the enzyme’s activity is promoted by blocking of its active center by an inhibitor, which disables it from binding with the reaction substrate (Taofiq et al. 2016).

**Table 2:** Antityrosinase activity of the extracts obtained from the mycelial cultures of *Ganoderma applanatum*, *Laetiporus sulphureus*, and *Trametes versicolor*

Concentration [µg/mL]	Inhibition of tyrosinase activity (%)			
	*Ganoderma applanatum*	*Laetiporus sulphureus*	*Trametes versicolor*	Positive control
3000	66.74 ± 0.24***	80.87 ± 0.27***	95.48 ± 0.37^ ns^	98.25 ± 0.34
2000	50.39 ± 0.26***	80.18 ± 0.31^ ns^	91.75 ± 0.24***	79.24 ± 0.65
1000	41.43 ± 0.26***	78.74 ± 0.43***	40.78 ± 0.27***	63.80 ± 0.05
700	23.65 ± 0.106***	46.25 ± 0.52***	35.80 ± 0.68***	55.65 ± 0.20
500	20.76 ± 0.24***	43.71 ± 0.17***	22.01 ± 0.04***	42.68 ± 0.17
300	15.70 ± 0.13***	36.69 ± 0.21***	19.2 ± 0.12***	30.06 ± 0.02
200	7.62 ± 0.21***	13.77 ± 0.21***	17.73 ± 0.16***	20.69 ± 0.15
100	7.60 ± 0.32***	12.57 ± 0.21***	15.51 ± 0.17***	13.65 ± 0.06
0	0.00^ ns^	0.00 ns	0.00 ns	0.00
IC_50_ [µg/mL]	1896	593.5	1239	644.98

Positive control: kojic acid

Data are presented as the mean ± SD (standard deviation); n = 3 repetitions. Anova test was used to reveal the differences between paired groups of elements in rows

*Differences statistically significant for p < 0.05 vs. positive control

**Differences statistically significant for p < 0.01 vs. positive control

***Differences statistically significant for p < 0.001 vs. positive control

*ns* not statistically significant

(Statsoft STATISTICA)

Effective tyrosinase inhibitors, such as hydroquinone or arbutin, are regrettably not devoid of undesirable effects. For instance, hydroquinone exhibits cytotoxic effects on melanocytes and is suspected to have a mutagenic effect on mammalian cells (Migas and Borys 2012). Fortunately, mushrooms can synthesize numerous compounds that are capable of inhibiting tyrosinase. These include kojic acid, azelaic acid, or 3,4-dihydroxybenzaldehyde. In the conducted study, the capacity of kojic acid accumulation was analyzed in the mycelial cultures. The compound was determined in the extracts at a level of 0.014 and 0.023 mg/100 g DW in *G. applanatum* and *L*. *sulphureus*, respectively (Table 1).

Kojic acid is the most precisely studied inhibitor of the discussed enzyme and is obtained from *Aspergillus* fungi growing on corn kernels. Due to its good water solubility, it is extensively used in cosmetic preparations. Apart from the depigmentation properties, kojic acid also exhibits antibacterial activity and prevents the formation of free radicals. The preparations containing this component not only lighten up discolorations but also have antiwrinkle and moisturizing effects. Moreover, kojic acid inhibits oxygen absorption, which is indispensable for enzymatic browning (Noh et al. 2009).

The next analysis aimed at assessing the inhibitory effect of the extracts obtained from the mycelial culture biomass of the studied species on hyaluronidase activity. The experiment demonstrated that the extracts showed a differential inhibiting effect on the enzyme. The extract of *G. applanatum* was practically devoid of this activity, while the extracts of *L. sulphureus* and *T. versicolor* showed dose-dependent hyaluronidase inhibition. Particularly impressive results were obtained for the *L. sulphureus* extract as it showed considerably higher hyaluronidase inhibition in comparison to the remaining analyzed samples. The analysis of *L. sulphureus* extract at 1 mg, and higher concentrations, did not show any statistically significant differences in the activity between the extract and the used reference inhibitor (quercetin). The results of the analysis of hyaluronidase-inhibiting effect of the extracts are presented in Table 3. Hyaluronidase is an enzyme belonging to the hydrolase group. It depolymerizes hyaluronic acid, which is the main intercellular link in the vertebrate cells playing a key role in retaining proper skin function.

**Table 3:** Antihyaluronidase activity of the extracts obtained from the mycelial cultures of *Ganoderma applanatum*, *Laetiporus sulphureus*, and *Trametes versicolor*

Concentration [µg/mL]	Inhibition of hyaluronidase activity (%)			
	*Ganoderma applanatum*	*Laetiporus sulphureus*	*Trametes versicolor*	Positive control
3000	1.56 ± 1.02***	97.76 ± 1,27^ ns^	24.28 ± 0.63***	96.57 ± 1.33
2000	0.67 ± 0.67***	93.50 ± 0.36^ ns^	16.67 ± 0.49***	95.27 ± 1.39
1000	0.23 ± 0.39***	87.76 ± 6.49^ ns^	6.99 ± 0.70***	91.11 ± 1.50
700	0.00 ± 0.00***	34.18 ± 2.90***	2.86 ± 0.45***	80.56 ± 0.75
500	0.00 ± 0.00***	6.22 ± 0.77***	2.17 ± 1.92***	38.84 ± 1.33
300	0.00 ± 0.00***	2.55 ± 1.10***	0.00 ± 0.00***	21.86 ± 4.04
200	0.00 ± 0.00***	0.43 ± 0.75***	0.00 ± 0.00***	13.86 ± 2.70
100	0.00 ± 0.00*	0.00 ± 0.00*	0.00 ± 0.00*	4.39 ± 0.88
0	0.00 ± 0.00 ns	0.00 ± 0.00 ns	0.00 ± 0.00 ns	0.00 ± 0.00
IC_50_ [µg/mL]	> 3000	760.01	> 3000	509.79

Positive control: quercetin (dihydrate)

Data are presented as the mean ± SD (standard deviation); n = 3 repetitions. Anova test was used to reveal the differences between paired groups of elements in rows

*Differences statistically significant for p < 0.05 vs. positive control

**Differences statistically significant for p < 0.01 vs. positive control

***Differences statistically significant for p < 0.001 vs. positive control

*ns* not statistically significant

(Statsoft STATISTICA)

Hyaluronic acid also acts on the walls of blood vessels by increasing their permeability. In medicine, it is used, among others, to improve the permeability of connective tissues (Stern and Jedrzejas 2006). Many natural and synthetic substances have an inhibiting effect on the activity of the hyaluronidase group of enzymes, which are involved in the decomposition of hyaluronic acid. In the human skin, hyaluronic acid binds to water. It is abundant in young skin, and thus guarantees flexibility. With age, the amount of this acid is reduced, and the skin loses its capacity to bind water, due to which wrinkles appear. Therefore, identifying natural sources of hyaluronidase inhibitors that could contribute to the development of new preparations seems justifiable (Taofiq et al. 2016).

### SPF determination

The results of SPF determination in the extracts are shown in Table 4. The SPF values of the studied extracts were found to range from 2.17 (*T. versicolor*) to 9.03 (*G. applanatum*). The extract *G. applanatum* had the highest SPF value (~ 9) and could thus act as a valuable component of formulation that provides sun protection as well as a natural source of antioxidants.

**Table 4:** Results of SPF determination

Mycelial cultures	Wavelength (nm)							SPF
	290	295	300	305	310	315	320	
Absorbance								
*T. versicolor*	0.2352	0.2316	0.2244	0.2172	0.2064	0.1992	0.2064	2.17
*L. sulpureus*	0.3367	0.3367	0.3367	0.3323	0.3237	0.3108	0.3065	3.30
*G. applanatum*	0.9525	0.9299	0.8998	0.8546	0.9827	0.8998	0.8395	9.03

(n = 3)

Lohézic-Le Dévéh et al. (2013) examined the lichen extracts for their photoprotective activities. Among the studied extracts, the extract of *Lasallia pustulata* exhibited an SPF value of 5.52 (Lohézic-Le Dévéhat et al. 2013). The SPF value obtained for *G. applanatum* suggests that its extract could be considered as a potential UV filter.

## Conclusion

The biomass from the mycelial cultures of the studied species exhibited the capacity of endogenous accumulation of compounds with potential applicability for treating skin lesions. The mentioned metabolites are characterized by the following properties: antioxidative, antimicrobial, astringent, antisweat, UV protection, epidermis-exfoliating (phenolic acids), moisturizing, protective, lubricating, emulsifying, healing (sterols), pigmenting (indole derivatives), and whitening (kojic acid). In addition, the extracts from the mycelial cultures exhibited valuable biological effects that are desirable in cosmetology. The extract of *L. sulphureus* showed dose-dependent hyaluronidase inhibition. Tyrosinase-inhibitory activity was observed in all the examined extracts. In the future, we have planned to study the impact of factors (e.g. precursors of metabolic pathways) which increase the accumulation of skin-beneficial compounds in in vitro cultures.
